# Comparison of Neurological Function in Males and Females from Two Substrains of C57BL/6 Mice

**DOI:** 10.3390/toxics3010001

**Published:** 2014-12-25

**Authors:** Amy Ashworth, Mark E. Bardgett, Jocelyn Fowler, Helen Garber, Molly Griffith, Christine Perdan Curran

**Affiliations:** 1 Department of Biological Sciences, Northern Kentucky University, SC344 Nunn Dr, Highland Heights, KY 41099, USA; E-Mails: ashwortha1@nku.edu (A.A.); jocelyncfowler@yahoo.com (J.F.); hfgmew01@aol.com (H.G.); 2 Department of Psychological Science, Northern Kentucky University, MP 359 Nunn Dr, Highland Heights, KY 41099, USA; E-Mails: bardgettm@nku.edu (M.E.B.); molly.griffith@cchmc.org (M.G.); 3 Division of Neurology, Department of Pediatrics, Cincinnati Children’s Hospital Medical Center, 3333 Burnett Avenue, Cincinnati, OH 45229, USA; 4 The Royal (Dick) School of Veterinary Studies, the University of Edinburgh, Edinburgh EH25 9RG, UK

**Keywords:** C57BL/6, substrain, sex differences, behavior, neurobiology, genetics

## Abstract

The C57BL/6 (B6) mouse is the background strain most frequently used for genetically-modified mice. Previous studies have found significant behavioral and genetic differences between the B6J (The Jackson Laboratory) and B6N substrains (National Institutes of Health); however, most studies employed only male mice. We performed a comprehensive battery of motor function and learning and memory tests on male and female mice from both substrains. The B6N male mice had greater improvement in the rotarod test. In contrast, B6J female mice had longer latencies to falling from the rotarod. In the Morris water maze (MWM), B6J males had significantly shorter latencies to finding the hidden platform. However, B6N females had significantly shorter path lengths in the reversal and shifted-reduced phases. In open field locomotor activity, B6J males had higher activity levels, whereas B6N females took longer to habituate. In the fear conditioning test, B6N males had a significantly longer time freezing in the new context compared with B6J males, but no significant differences were found in contextual or cued tests. In summary, our findings demonstrate the importance of testing both males and females in neurobehavioral studies. Both factors (sex and substrain) must be taken into account when designing developmental neurotoxicology studies.

## 1. Introduction

Many neurological disorders are linked to genetic defects or gene-environment interactions, so the study of genetically-modified mice is invaluable in identifying causal mutations and understanding genetic susceptibility to environmental insults. Our recent search of PubMed turned up more than 33,000 papers on mouse brain development, demonstrating the importance of mouse models in neuroscience and developmental neurotoxicity. The existence of numerous inbred strains of mice provides an advantage for genetic studies, because a common genetic background makes it easier to discern the role of individual genes and the impact of allelic variation [1,2,3]. Inbreeding also reduces behavioral variability within a strain, although it does increase variability among strains. For example, BALb/c, C57BL/6, A/J, and BTBR all have distinct behavioral phenotypes [2,4,5].

The C57BL/6 (B6) mouse was the first mouse strain to have its genome sequenced [6], and it is the most commonly-used background strain for genetically-modified mice [7,8]. The B6 strain originated in 1921, and a colony was moved to The Jackson Laboratory (Bar Harbor, ME), which gave rise to the B6J substrain. The founders of the C57BL/6N (B6N) line were isolated from the Jackson substrain at the National Institutes of Health in 1951 [9]. These two substrains are indistinguishable from their exterior phenotype; however, genetic drift over the decades produced quantifiable allelic differences. These include a null mutation in the nicotinamide nucleotide transhydrogenase (*Nnt*) gene, which reduces insulin secretion in B6J, whereas B6N have the wild-type allele and are unaffected [10]. Of interest to neurological studies, a subpopulation (B6JOlaHsd) was found to have a mutation in the alpha synuclein gene (*Snca*) [11], and B6N mice are homozygous for a mutation in the cytoplasmic *FMR1* interacting protein 2 gene *Cyfip*, resulting in an attenuated response to cocaine and methamphetamine [12]. Mekada *et al.* [13] and Zurita *et al.* [14] used an Illumina platform to identify 11 additional single-nucleotide polymorphisms (SNPs), further distinguishing the B6J and B6N substrains.

B6 substrains also differ in behavioral phenotype based on reported results from rotarod, fear conditioning, pain sensitivity, open field locomotor activity, elevated plus maze and prepulse inhibition [7,15,16]. However, these studies only used male mice. Consistent with recent recommendations by the U.S. National Institutes of Health [17], we performed a battery of neurobehavioral tests on male and female B6J and B6N mice in sequential experiments to see if the effects reported in male mice would be replicated in the females. We also purchased all mice from the same vendor to avoid confounding by source [15,18].

Our behavioral battery was selected to be representative of the most commonly-used tests for learning and memory, activity and motor function. For replication, we included two previously-reported tests (open field locomotor activity and fear conditioning). The learning and memory tests were selected to expand upon previous findings and for their ability to identify deficits in spatial, non-spatial and fear learning (*i.e.*, deficits in the medial prefrontal cortex, hippocampus or amygdala) [3,19].

## 2. Experimental Section

### 2.1. Animals and Handling

Nine-week-old C57BL/6J (B6J) and C57BL/6N (B6N) mice were purchased from The Jackson Laboratory (Bar Harbor, ME, USA). All mice were group housed, four per cage, by sex and substrain, in standard static micro-isolator polysulfone shoebox cages with corn cob bedding and one nestlet for enrichment. Water and lab chow (Purina 5015) were provided *ad libitum*. Cages were changed once per week. During experiments, cages were changed following behavioral testing to avoid confounding by stress [20]. No significant adverse health issues were reported in the vivarium during the period when the experiments were performed (e.g., fungal, viral, bacterial or parasitic infections).

Behavior experiments began after an acclimation period of one week. All protocols were approved by the Northern Kentucky University (NKU) Institutional Animal Care and Use Committee in accordance with the Guide for the Care and Use of Laboratory Animals [21]. The experiment began with a sample size of 20 per group. Two B6N males were removed from the study after suffering fight wounds, leaving one cage with two mice. The final group size was *n* = 18 for B6N males and *n* = 20 for all other groups. In keeping with the recommendations of Sorge *et al.* [22], we report that all experiments were conducted entirely by female investigators.

All experiments were conducted during the same 4-h time block during the light portion of a 12 h:12 h light-dark cycle. Animals received only one test per day. The order of experiments is the same as the order in which we report them: open field locomotor, rotarod, novel object recognition, Morris water maze and conditioned fear. The general order is from least stressful to most stressful. Mice were tested in the same order for all tests. The initial cohort consisted of 40 males. A follow-up experiment was conducted with 40 females. All equipment was sanitized and deodorized between test animals using 70% ethanol. The Morris water maze was drained and cleaned weekly.

### 2.2. Open Field Locomotor Activity

Mice were placed in arenas (41 cm × 41 cm) that contained a 16 × 16 LED photobeam array in the *x-* and *y-*planes, with a third row of photobeams in the *z-*plane to detect rearing [23]. Locomotor activity was recorded for 1 h in 5-min intervals using Photobeam Activity System software (San Diego Instruments, San Diego, CA, USA). Activity was measured as total beam breaks.

### 2.3. Rotarod

The rotarod (IITC Life Science, Woodland Hills, CA, USA) was used to assess cerebellar function. For stationary training, each mouse was placed on the stationary rotarod facing the back wall of the lane for a maximum of 60 s with a 15-s intertrial interval. To proceed to the second phase of training, mice passed three consecutive trials without falling. On the second day of training, the rotarod was set at a constant speed of 2 revolutions per minute (rpm), and each trial lasted a maximum of 60 s. The intertrial interval was 15 s. To proceed to acceleration testing, mice passed three consecutive trials without falling. All mice successfully passed each training phase. During the acceleration test phase, the rotarod accelerated from 1 to 20 rpm over 180 s with a maximum trial length of 300 s. There were three trials per day with a 10-min intertrial interval and five days of testing. The latency to fall was compared. A fall was defined as physically falling off the rotarod or slipping in a way that only the front paws were still grasping the rod.

### 2.4. Novel Object Recognition

For the first two training days, mice were placed in a plastic, circular arena (61 cm in diameter) for 10 min of acclimation. For the next two acclimation days, mice were placed in the arena with two practice objects placed equidistantly from each other for 10 min each day. The practice objects were distinctly different from the objects used in testing and used to reduce neophobia. All objects were heavy enough that the mice could not move them and were constructed out of sturdy glass or ceramic materials. Testing took place on Day 5 and was monitored by the same observer using a remote camera. Two identical, familiarization objects were placed in the arena, and mice were given 10 min to accumulate 30 s of exploration of the objects. The percent of time exploring each familiarization object was recorded to determine if there was a place preference or left-right bias. Exploration was defined as being within 1 cm of the object and oriented towards the object. One hour after the familiarization phase, mice were placed in the arena with an identical copy of the familiar object and a novel object. The percent of exploration time of the novel object out of the total 30 s was recorded. Test and familiarization objects were counterbalanced to avoid object preference [24].

### 2.5. Morris Water Maze

Four phases were used for the Morris water maze (Table 1) with a 122-cm diameter pool filled with non-toxic white tempera paint and kept at 22 °C (±1 °C), which are optimal conditions for testing B6 mice [25]. In the cued phase, mice were placed facing the wall and given 60 s to find a submerged 10-cm diameter escape platform that had an orange ball attached to a 10 cm-tall pole in the middle of the platform. Day 1 of the cued phase consisted of 6 trials per day with fixed start and platform locations. On Days 2–6, mice received 2 trials per day with random start and platform locations. During the cued platform phase, curtains surrounding the pool were closed, but opened during the hidden platform phases (acquisition, reversal and shift-reduced) to reveal distal visual cues. The escape platform was submerged 1 cm under the water. For each hidden platform phase, the diameter of the platform was progressively smaller (10 cm, 7 cm and 5 cm) and the location was moved for each week of testing. There were six days of testing and four trials per day. Each trial had a maximum of 60 s and a 15-s intertrial interval. Mice failing to find the platform in 60 s were placed on the platform for 10 s. On Day 7, mice received one 30-s probe trial with no platform. Path length, latency to reach the escape platform and swim speed were recorded using ANY-maze™ software (Stoelting, Inc., Wood Dale, IL, USA). Platform crossings and time in the target quadrant were compared for the probe trials.

**Table 1:** Platform positions and cues for the Morris water maze.

Schedule	Platform Size	Platform Location	Cues
Week 1-Cued	10 cm	Varies each day	Proximal cue on platform
Week 2-Acquistion	10 cm	Southwest	Distal cues on walls
Week 3-Reversal	7 cm	Northeast	Distal cues on walls
Week 4-Shift-reduced	5 cm	Northwest	Distal cues on walls

### 2.6. Fear Conditioning

The fear conditioning protocol was based on a protocol described in Bardgett *et al.* [26] and Yin *et al.* [27]. Mice were placed in an operant chamber in a sound-attenuating apparatus (Med-Associates, St. Albans, VT, USA) for 5 min on the training day. A single observer scored all behavior. Freezing behavior, defined as no movement excluding normal respiratory movement, was sampled for 2 s every 10 s. An 80-dB tone at 2800 Hz was presented for 20 s after 3 min. Mice received a 0.8-mA continuous foot shock at the last second of the tone. The tone and shock pairing was repeated every minute for the last 2 min. Mice were removed from the test cage 40 s after the third shock and returned to their home cage. The presentation of the conditioned and unconditioned stimuli was automated using MED-PC software (Med-Associates). Animals were returned to the original operant chamber 48 h after training for contextual fear conditioning. For 8 min, freezing behavior was recorded every 10 s by an observer. Twenty-four hours after the contextual fear conditioning phase, animals were placed in a white polycarbonate cage approximately the same size as the original operant chamber to test for cued fear conditioning. A speaker was placed on top of the testing cage, which emitted the same tone used on the training day. Freezing behavior was recorded every 10 s in the test cage. After 2 min, the same auditory cue was emitted continuously for the last 8 min. Any freezing behavior during this time represented the conditioned response to the auditory cue. Fear conditioning tests were only performed on male mice, because the same observer was not available to test the female mice.

### 2.7. Statistical Analysis

Behavioral data for open field locomotor activity, rotarod and the Morris water maze were analyzed using SAS 9.2 (SAS Institute, Cary, NC, USA) ProcMixed mixed models analyses of variance (ANOVA) with a repeated measures design. Data from males and females were analyzed separately, because the experiments were conducted sequentially. Swim speed was used as a covariate (ANCOVA) when analyzing the latency to finding the platform in the Morris water maze. In all cases, the best-fit model was auto-regressive. When significant differences were found, *post hoc* analyses were done using tests that correct for multiple comparisons. When only two groups were being compared without repeated measures, SPSS 19.0 (IBM, Armonk, NY, USA) was used to conduct an independent samples *t*-test. All data are presented as the least square means (ANOVAs and ANCOVAs) or arithmetic means (*t*-tests) ± the standard error of the mean (SEM).

## 3. Results

### 3.1. Open Field Locomotor Activity

**Figure 1:**
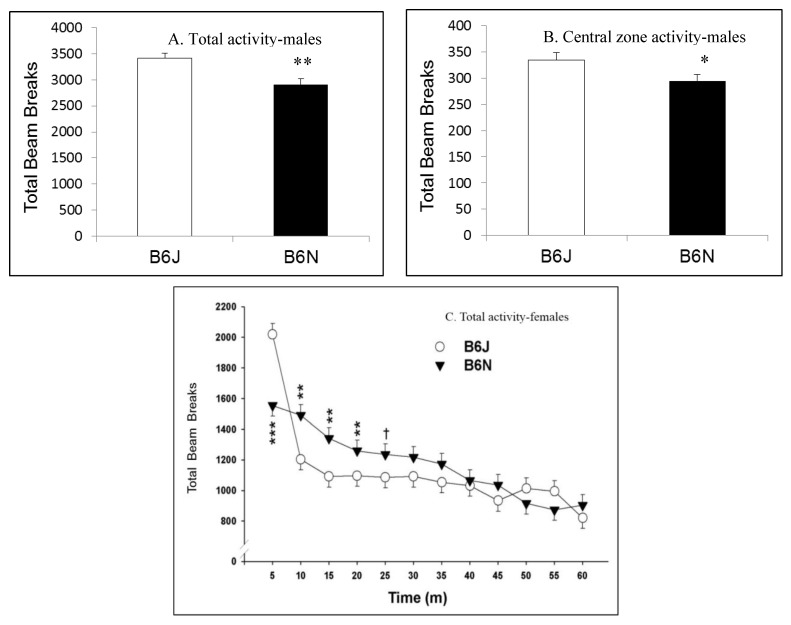
(**A**–**C**) Open field locomotor. There was a main effect of genotype for males (*p* < 0.01) and a gene × interval interaction for females (*F*_11,418_ = 4.97; *p* < 0.001). The B6J males had higher total activity throughout the test session compared with B6N males (*p* < 0.01). The B6J males also had greater activity in the central zone compared with the B6N males (*p* < 0.05). During the first 5-min interval, B6J females were significantly more active than B6N females (*p* < 0.001). The B6N female mice were significantly more active during the next three intervals (*p* < 0.01), and there was a trend for significance (*p* < 0.1) in the fifth interval. Data are the least square means ± S.E.M. * *p* < 0.5, ** *p* < 0.01, *** *p* < 0.001.

C57BL/6J male mice were significantly more active than C57BL/6N males when analyzing total beam breaks (Figure 1A) and activity in the central zone (Figure 1B). A different pattern was observed in female mice (Figure 1C). B6J females had higher activity in the first 5-min interval, but B6N females were more active during the next 20 min. This suggests that B6N females took longer to habituate to the test apparatus than the B6J females.

### 3.2. Rotarod

**Figure 2:**
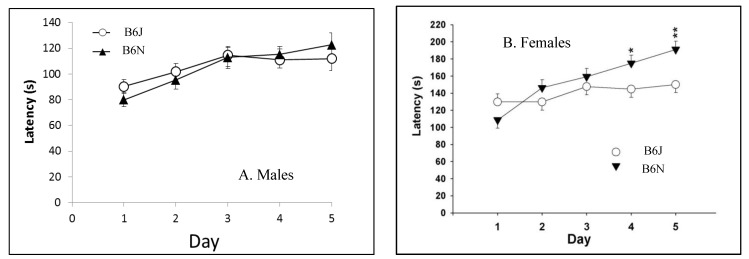
(**A**,**B**) Rotarod. B6N males showed a greater percent increase in the latency to falling from Day 1 to Day 5 of testing compared with B6J males, and there was a significant effect of day (F_4,144_ = 12.75; *p* < 0.0001). B6N female mice also showed a greater percent improvement in the latency to falling over the five days of testing and significantly longer latencies to falling on Days 4 and 5. * *p* < 0.05, ** *p* < 0.01. Data are the least square means ± S.E.M.

There were no significant differences on any of the five days of testing when B6J and B6N males were tested; however, B6N males had a greater percent increase in the latency to falling over the five-day test compared with B6J males (Figure 2A). B6N males improved 53% by Day 5, whereas B6J males only improved 24%. For female mice, there was a significant gene × day interaction (*F*_4,122_ = 4.97; *p* = 0.001) (Figure 2B). B6N females had a 76% increase in the latency to falling across the five days of testing compared with a 16% increase for B6J females.

**Figure 3:**
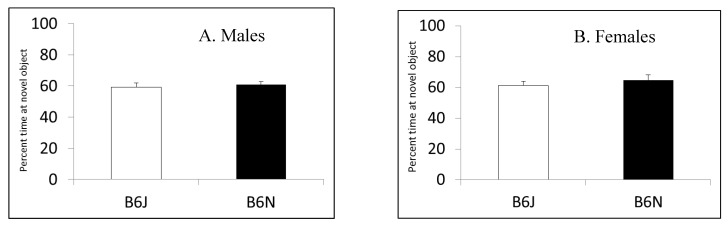
(**A**,**B**) Novel object recognition. There were no significant differences in the percentage of time spent exploring the novel object in male mice or female mice (*p* > 0.05). Data are the means ± S.E.M.

### 3.3. Novel Object Recognition

There were no significant differences in the percent of time exploring either of the familiarization objects (left *vs*. right object). During the test phase, there were no significant differences when comparing substrains B6 male (Figure 3A) or female (Figure 3B) performance. All groups of mice spent more time exploring the novel object in comparison to the familiar object, indicating that non-spatial learning and memory was normal.

### 3.4. Morris Water Maze

**Figure 4:**
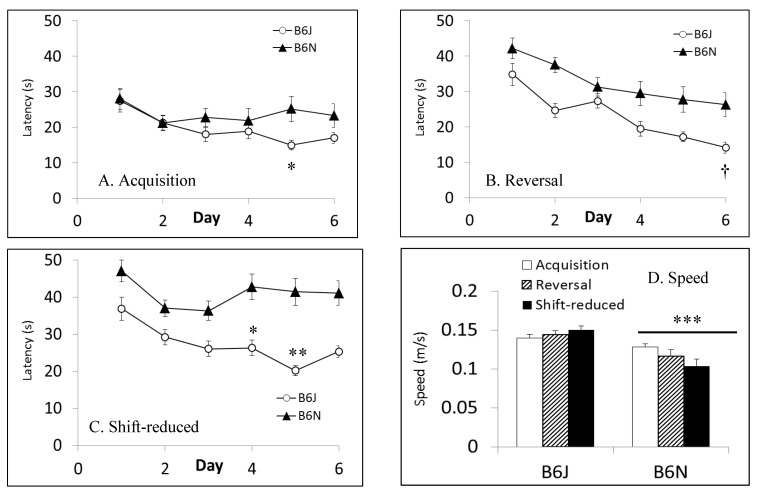
(**A**–**D**) Morris water maze latency: males. B6J males had shorter latencies to escaping. Swim speed was significantly different in all three phases of hidden platform testing. ^†^ *p* < 0.1, * *p* < 0.05, ** *p* < 0.01, *** *p* < 0.001. Data are the least square means ± S.E.M.

**Figure 5:**
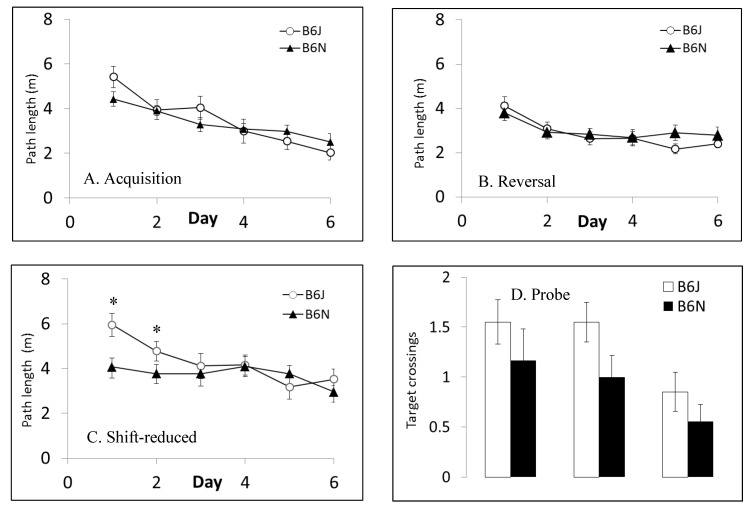
(**A**–**D**) Morris water maze path length: males. When analyzing path length, there were no significant differences in the acquisition or reversal phases, but a significant difference on Days 1–2 in the shift-reduced phase with shorter path lengths in B6N males. The number of target crossings during the probe trials was not significantly different for males in any of the three phases. * *p* < 0.5. Data are the least square means ± S.E.M.

There were no significant differences in the cued platform phase for male mice (data not shown). We found significant differences in all three phases of hidden platform testing (Figure 4A–C). There was a main effect of day in all three phases (*p* < 0.001) and a gene × day interaction. B6J males had significantly shorter latencies on Day 5 of acquisition testing (*F*_1,182_ = 4.29; *p* < 0.05), a trend toward significance on Day 6 of reversal testing (*F*_1,148_ = 2.76; *p* < 0.1) and significantly shorter latencies on Days 4 (*F*_1,143_ = 3.77; *p* < 0.05) and 5 (*F*_1,143_ = 7.29; *p* < 0.01) of shift-reduced testing after correcting for speed as a covariate. B6N males had shorter path lengths in the shift-reduced phase (Figure 5A–C). We also found significant differences in swim speed, with B6N males swimming more slowly than B6J males (Figure 4D); however, latency differences remained significant even after correcting for this covariate. No differences were found in the probe trials for males (Figure 5D).

**Figure 6:**
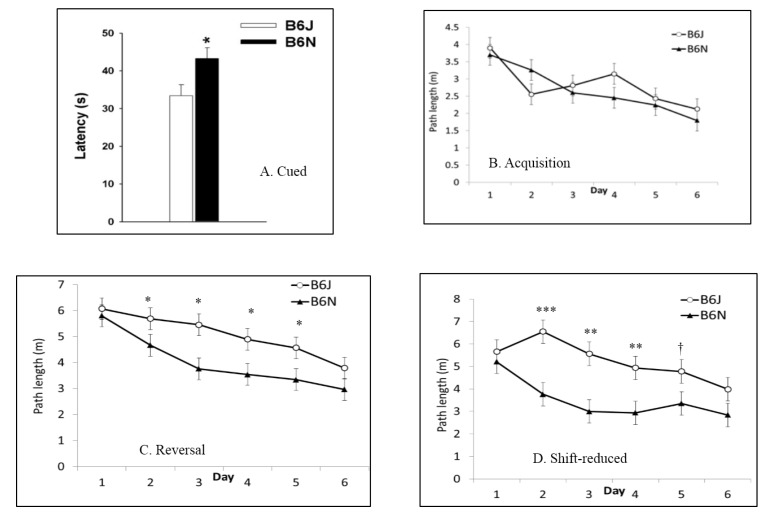
(**A**–**D**) Morris water maze: females. B6J females had significantly shorter latencies to finding the platform on Day 1 of the cued phase (*p* < 0.05). Path lengths were significantly different on Days 2–5 of the reversal phase and Days 2–4 of the shift-reduced phase with a trend on Day 5. ^†^ *p* < 0.1, * *p* < 0.05, ** *p* < 0.01, *** *p* < 0.001. Data are the least square means ± S.E.M.

**Figure 7:**
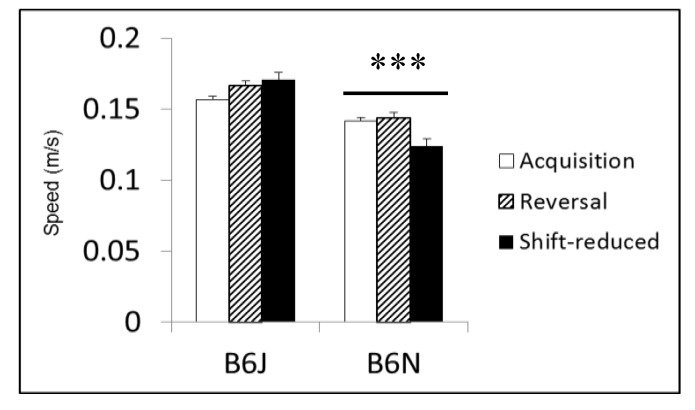
Morris water maze swim speed: females. B6J females swam significantly faster than B6N females in all three hidden platform phases. *** *p* < 0.001. Data are the least square means ± S.E.M.

B6J females had shorter latencies on Day 1 of cued platform testing (Figure 6A) compared with B6N females, but there were no other significant differences in latency to find the platform on Days 2–6 in cued or in any of the three hidden platform phases (data not shown). There was a significant main effect of day in all four phases (*p* < 0.001). When path lengths were compared, there were no significant difference during the acquisition phase (Figure 6B). However, B6N females had significantly shorter path lengths during the more challenging reversal and shift-reduced phases (*F*_1,122_ = 14.34; *p* < 0.001) (Figure 6C,D). B6N females had significantly more platform crossings in the acquisition probe trial (*F*_1,38_ = 16.21; *p* < 0.0001), but there were no significant differences in the probe trials for reversal and shift-reduced (data not shown). Swim speeds were similar in females, with B6J mice swimming faster than B6N females (Figure 7).

### 3.5. Fear Conditioning

**Figure 8:**
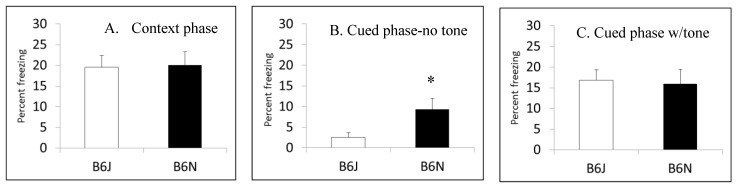
(**A**–**C**) There were no significant differences in contextual or cued fear conditioning. However, B6N males spent a significantly longer time freezing than the B6J males when placed in the novel cage used for cued fear conditioning before the tone (cue) was presented. * *p* < 0.05. Data are the least square means ± S.E.M.

There was no significant difference in the percent of time spent freezing between the B6J and the B6N males during the training phase (data not shown) or the context phase test (Figure 8A). Prior to the onset of the tone in the cued phase, the B6N males spent a significantly longer time freezing (Figure 8B) than the B6J males (*p* < 0.05). There was no significant difference between the percent time freezing between the two substrains during the cued phase (Figure 8C).

In summary, we found significant behavioral differences in two substrains of C57BL/6 mice purchased from the same vendor and have demonstrated that patterns of behavior in male and female mice from the B6J and B6N substrains are not identical (Table 2).

**Table 2:** Summary of significant findings.

Behavior Test	Function	Males	Females
Open field locomotor	Baseline activity	Higher activity in B6J	Slower habituation in B6N
Rotarod	Balance and coordination	Greater improvement in B6N over five test days	Significantly longer latencies in B6N; greater improvement in B6N over five tests days
Novel object recognition	Non-spatial learning and memory	No significant differences	No significant differences
Morris water maze	Spatial learning and memory	Shorter latencies to escape in B6J; faster swim speeds in B6J; shorter path lengths in shift-reduced in B6N	Shorter path lengths in reversal and shift-reduced in B6N; faster swim speeds in B6J
Fear conditioning	Fear learning	More freezing in cued phase before tone by B6N	Not tested

## 4. Discussion

After sequentially testing male and female mice of the B6J and B6N substrains, we found that B6J males were more active in open field locomotor activity compared with B6N males, replicating the findings of Matsuo *et al.* [7]. B6J females were more active during the first 5-min interval, but habituated to the chambers more quickly than B6N females. Bothe *et al.* [28] examined locomotor activity during the dark cycle in male and female B6J and C57BL/6NTac mice, reporting higher activity in B6J females compared with B6J males and no sex differences for the B6Ntac substrain. All four groups showed normal habituation. Further work is needed to explore the effects of circadian rhythms in the two substrains.

B6N males showed greater improvement (53 *vs*. 24 percent) over five days of rotarod testing, but the differences in latency to falling were not significant on any individual test day. In contrast, B6N females had significantly longer latencies to falling on the final two days of testing and improved 76% over the five days of testing compared with only 16% improvement in B6J females. Interestingly, Bothe *et al.* [28] also reported better performance in females compared with male B6 mice and greater improvement over three days of testing in C57BL/6NTac mice using a top speed of 15 rpm. In contrast, both Matsuo *et al.* [7] and Bryant *et al.* [15] reported longer latencies for B6J males using a top speed of 40 and 60 rpm, respectively. This suggests that the difficulty of the test and the length of testing can alter the results for the rotarod.

There were no significant differences in novel object recognition, indicating no difference in hippocampal-dependent, non-spatial visual learning and memory. B6J males had shorter latencies in the Morris water maze in the more difficult reversal and shift-reduced phases after correcting for swim speed; however, B6N males had shorter path lengths on the first two days of the shift-reduced phase. B6J females had shorter latencies to finding the platform on the first day of the cued platform, but B6N females had shorter path lengths in both the reversal and shift-reduced hidden platform phases and more platform crossings in the acquisition probe trial. Both male and female B6J mice had significantly faster swim speeds than their B6N counterparts. This is consistent with their initial higher activity in locomotor activity and previous findings [7]. Our findings differ from those of Clapcote and Roder [29], who compared females from eight strains of mice, including B6J and B6NTac, and reported longer latencies and cumulative distance in the acquisition and reversal phases in B6NTac females and no difference in swim speed. This suggests the source of the mice could impact results in tests of spatial learning and memory, since our B6N mice came from a different vendor. Alternatively, differences in handling are also known to affect the performance of B6 mice in the Morris water maze [30]. 

We found no difference in contextual or cued fear conditioning, although B6J males showed less freezing when exposed to the new chamber used in the cued test compared with B6N males. This is similar to the findings of Bryant *et al.* [15], who reported significantly more freezing in B6N males during the contextual phase and significantly less freezing by B6J males during the altered context, but before the cue was given. Bryant also tested pain sensitivity using a hot plate and tail withdrawal test, reporting that B6J mice had shorter latencies to respond. This indicates that pain sensitivity is not the driving factor for the observed differences in fear conditioning.

Physiological differences across B6 substrains have been reported, as well as the genetic polymorphisms and behavioral differences noted earlier. These should also be of interest to those conducting neurobehavioral studies, because they could confound the results of tests requiring stamina, such as the rotarod, Morris water maze and the Porsolt forced swim test. Garcia-Menendez [31] reported differences between B6J and B6N males in response to cardiac overload, while Vaillant *et al.* [32] reported significantly greater cardiac output, cardiac power and heart rate in male B6NCrl compared with male B6J. Tests of visual acuity could be affected by rd8 mutations in the *Crb1* gene found in B6N mice [33], although all B6 mice are susceptible to ocular disorders, such as microphthalmia [34].

Metabolic differences could explain the differences in the rates of alopecia and skin lesions in B6 substrains. Sundberg *et al.* [35] found that retinoic acid metabolism was upregulated in B6J and B6Tac substrains, but not in the resistant B6Crl or B6NCr substrains. Differences in the background substrain changed the outcome in a study on acetaminophen toxicity in JNK2 knockout mice [18]. Others have reported differences in airway responsiveness in B6 substrains ordered from different vendors, but at least some of the observed differences disappeared after an F1 generation was raised, suggesting that environmental factors were more important than genetic ones [36].

Although there are fewer studies on other strains of rodents, substrain differences consistently appear when other lines are compared. Examples include the 129 strain, which is frequently used as a source of embryonic stem cells [37,38]. Eisner-Dorman *et al.* [39] demonstrated that remnant 129 genes can influence behavioral phenotypes, even when mice are back-crossed extensively to a B6 background. The 129S6/SvEv substrain is important as a potential mode for schizophrenia, because there is a spontaneous deletion in the *DISC1* gene (disrupted in schizophrenia-1) [40]. Gómez-Sintes *et al.* [41] found that a 129-B6J DISC1^Del^ hybrid produced a more robust phenotype than mice with the deletion on a pure B6 background. Therefore, in some cases, increased genetic variability is beneficial to neurobehavioral research and the identification of gene-gene interactions.

Ultimately, the value of mouse models in developmental neurotoxicology studies will be realized when we are able to identify allelic differences and gene-environment interactions that alter normal neurodevelopment and neurological function in humans. The promise of these studies remains high, but as Kiselycznyk and Holmes [42] correctly noted, both epistatic interactions and penetrance can be affected by subtle genetic differences across substrains of mice. A case in point is the recent finding of SNPs in the BDNF gene that impair the effectiveness of anxiety treatments. Unfortunately, Soliman *et al.* [43] did not report the substrain nor vendor for the B6 mice used in those studies. Our work and that of others [2,5,7,15,28] strongly suggest that this information will be essential to replicating and interpreting data from studies using inbred mice, whether those mice are B6 or from another widely-used strain, such as 129 [37,38].

## 5. Conclusions

The behavioral phenotypes of B6J and B6N mice are not identical, and sex differences can further confound the interpretation of behavioral tests. Therefore, results from one sex should never be extrapolated to the other. Substrain choice should be taken into consideration when choosing the background strain for genetically-modified mice and reported to alert other investigators about potential gene-gene interactions. Inbred mouse strains and substrains will continue to be useful for identifying genetic pathways involved in neurological function and neurodevelopment. The availability of less expensive sequencing techniques increases the likelihood that informative SNPs in both coding and non-coding regions will be found to explain the observed phenotypic differences. Comparing substrains provides a unique opportunity to identify genetic differences associated with these behavioral differences, since the number of genetic differences is smaller than comparing one inbred strain to another or F1 hybrids. In summary, our findings demonstrate the importance of testing both males and females in neurobehavioral and neurotoxicological studies and highlight the importance of identifying the specific substrain and vendor used for all rodent experiments.
